# Prevalent and Incident Vertebral Deformities in Midlife Women: Results from the Study of Women’s Health Across the Nation (SWAN)

**DOI:** 10.1371/journal.pone.0162664

**Published:** 2016-09-22

**Authors:** Gail A. Greendale, Holly Wilhalme, Mei-Hua Huang, Jane A. Cauley, Arun S. Karlamangla

**Affiliations:** 1 David Geffen School of Medicine, University of California Los Angeles, Los Angeles, United States of America; 2 Department of Medicine Statistics Core, University of California Los Angeles, Los Angeles, United States of America; 3 Graduate School of Public Health, Department of Epidemiology, University of Pittsburgh, Pittsburgh, Pennsylvania, United States of America; University of Hull, UNITED KINGDOM

## Abstract

**Background:**

Vertebral fractures are the most common type of osteoporotic fracture among women, but estimates of their prevalence and incidence during middle-age are limited. The development of vertebral morphometry (VM) using dual energy X-ray absorptiometry (DXA) makes it more feasible to measure VM in large, longitudinal, observational studies. We conducted this study to: 1) contribute to the scant knowledge of the prevalence, incidence and risk factors for vertebral deformities in middle-aged women; and 2) to evaluate the performance of DXA-based VM measurement in a large, community based sample.

**Methods:**

The sample is derived from the Study of Women’s Health Across the Nation (SWAN), a multi-site, community-based, longitudinal cohort study of the MT. Using Hologic QDR 4500A instruments, we acquired initial VM measurements in 1446 women during calendar years 2004–2007; in 2012–2013, a follow-up VM was obtained in 1108. Annually, lumbar spine (LS) and femoral neck (FN) bone mineral density (BMD) were measured and participant characteristics were assessed with standardized instruments. Multivariable logistic regression models examined the relations between prevalent deformity and relevant characteristics. Analyses of characteristics associated with prevalent deformity were restricted to 824 women who had not taken bone active medications since SWAN baseline. We calculated incident deformity per person year (PY) of observation, standardized to 1000 person-years.

**Results:**

The cranial portion of the VM image yielded the lowest proportions of readable vertebrae: from T4 through T6, between 43% and 63% of vertebral bodies were evaluable. Greater BMI was associated with fewer readable levels (B = -0.088, p<0.0001). In the baseline sample of 1446 women, the prevalence of vertebral deformity was 3.2% (95% CI: 2.3, 4.1). The relative odds of deformity increased by 61% per SD decrement in baseline LS BMD (p = 0.02) and were 67% greater per SD decrement in baseline FN BMD (p = 0.04). Odds of prevalent deformity increased by 21% per year increment in age (p = 0.02). On average, 1108 women were followed for 6.8 years (SD 0.5 years, range 5.1–8.3 years) and we observed an incidence of 1.98 vertebral deformities per 1000 PY. In the longitudinal sample, 628 participants had never used bone active medications; their vertebral deformity incidence was 2.8 per 1000 PY.

**Conclusion:**

Prevalence of vertebral deformity in SWAN participants aged 50–60 years was low and lower bone density at the LS and FN was strongly related to greater risk of prevalent deformity. Only about half of the vertebral levels between T4-T6 could be adequately imaged by DXA. Greater BMI is associated with fewer readable vertebral levels.

## Introduction

While vertebral fractures (VF) are the most common type of osteoporotic fracture that occur during the female life course, relative frequencies of each type of osteoporotic fracture vary by age[1,2] Roughly two-thirds to three-fourths of vertebral fractures do not result in acute pain, making it difficult to obtain population-based estimates of VF prevalence and incidence challenging.[3,4] VF prevalence and incidence estimates require lumbar and thoracic X-rays, or more recently, vertebral morphometry obtained with dual energy X-ray absorptiometry (DXA), which has the advantage of very low radiation dose.[5,6] In observational studies, the term vertebral deformity, rather than fracture, is generally used to describe findings on X-rays or DXA morphometric analyses–because of challenges inherent in knowing whether the finding represents a vertebral fracture, an anatomic variant, or a gradual vertebral body shape change over time[7]

The *overall* prevalence of vertebral deformities in women over the age of 50 years of age is estimated at between 10% and 37%, but these averages are heavily influenced by deformity rates in women aged 65 years and older.[8–16] The prevalence of vertebral deformities in younger women, aged between 50 and 60 years, is substantively lower, at about 5%. However, the numbers of women on which estimates in this younger age stratum are based is low, pointing to the need for more information about vertebral deformity during middle age.[8–13, 16]

A better understanding of the prevalence of vertebral deformity in mid-life women is relevant to osteoporosis prevention efforts. Those with a prevalent deformity are about 5 times more likely to sustain an incident deformity over the course of one year and are also at double the risk of having other minimal-trauma fractures.[17] Asymptomatic vertebral deformities do count as prior fragility fractures in the FRAX® tool, the most commonly-used method of estimating absolute risk of future fractures (http://www.shef.ac.uk/FRAX). Vertebral deformities were also enrollment criteria and a primary endpoint for most randomized trials of osteoporosis therapies. Because the majority of vertebral fractures are asymptomatic, there is a growing interest in clinical use of vertebral morphometry assessment as part of DXA measurement to improve fracture risk stratification.[6] However, the cost-effectiveness of such a screening strategy depends on the prevalence of vertebral deformity in the population and the effectiveness of the screening assessment tool.

To contribute to the scant knowledge of the prevalence, incidence and risk factors for vertebral deformities in middle-aged women, and to gauge the performance of DXA-based VM screening in large, community based sample, the Study of Women’s Health Across the Nation (SWAN) conducted a vertebral morphometry study, the primary aims of which were to: 1) estimate prevalence and incidence of vertebral deformities in the SWAN sample; 2) explore whether prevalence and incidence varied by selected characteristics such as age or bone mineral density(BMD).

## Methods

### Study sample

**Fig 1:**
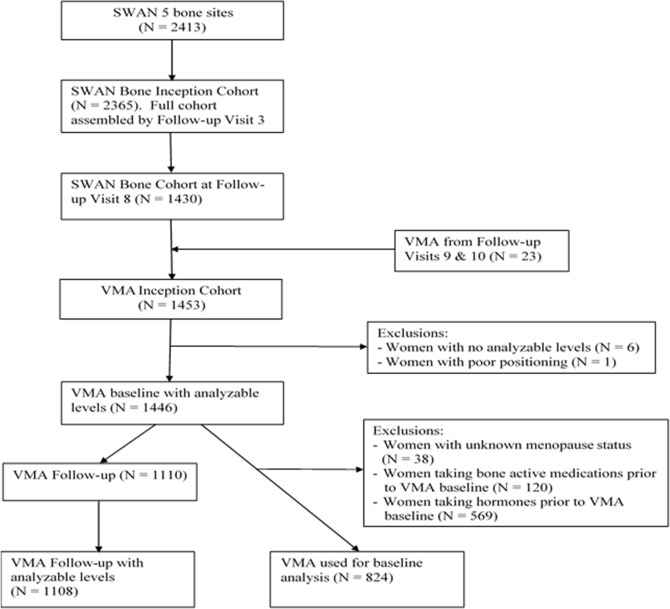
Baseline and follow-up sample derivation.

The study sample is derived from the bone study component of the Study Women’s Health Across the Nation (SWAN) [**Fig 1**]. The parent study, SWAN, is a multi-site, community-based, longitudinal cohort study of the MT.[18] SWAN eligibility criteria were: age at cohort between 42 and 52 years, intact uterus and at least one intact ovary, not using hormone therapy at the start of SWAN, at least one menstrual period in the 3 months before screening, and self-identification as a member of one of 5 eligible ethnic/racial groups. SWAN participants were enrolled at 7 sites: Boston, Chicago, Detroit, Pittsburgh, Los Angeles, Newark and Oakland (N = 3302). All sites enrolled White women; Boston, Chicago, Detroit, and Pittsburgh enrolled Black women and the remaining 3 sites enrolled Japanese, Hispanic and Chinese women, respectively. The SWAN bone study took place at 5 sites (Chicago and Newark did not participate); thus, the maximum number of potential enrollees in the bone study was 2413. Of these, 2365 women enrolled and 1430 remained in the bone cohort at SWAN follow-up visit 8, when the vertebral morphometry initial assessment began. We acquired initial (prevalent) vertebral morphometry measurements during SWAN follow-up visits 8 through 10 (calendar years 2004–2007); although obtained over the course of 4 calendar years, all were baseline vertebral morphometry scans. To assess incidence, we acquired a second vertebral morphometry scan during SWAN follow-up visit 13 (2012–2013). Vertebral morphometry was ascertained with Hologic QDR 4500A instruments (Hologic, Inc., Bedford, MA). During visits 8 through 10, 1446 women had an initial, usable, vertebral morphometry scan; this is the cross-sectional sample. Of the baseline sample, 1108 (76%) had a usable follow-up exam; these women comprise the longitudinal sample.

### Outcome

Lateral vertebral morphometry scans were read by a single expert research radiologist based at Synarc, Inc., using the Genant semi-quantitative method (Synarc, Inc., San Francisco, CA).[5,19] Starting with T4 and proceeding to L4, the radiologist first assessed whether each vertebral level was evaluable; for evaluable levels, she assigned each level a semi-quantitative fracture grade (SQ Grade); SQ grade is done by visual inspection, without direct vertebral measurement. Grade 0 is normal, no deformity. Grade 1 is mildly deformed (approximately 20–25% reduction in anterior, middle, and/or posterior height and a reduction of area 10–20%). Grade 2 is moderately deformed (approximately 25–40% reduction in any height and a reduction in area 20–40%. Grade 3 is severely deformed (approximately 40% reduction in any height and area). If a given vertebral level could not be evaluated, the radiologist reported the reason(s) such as: level not in scan, poor signal to noise ratio or overlying ribs (more than one reason could be cited for each level).

### Participant Characteristics

Standardized questionnaires assessed the following characteristics, of interest: age (years), education level (less than high school [HS], HS, more than HS, college, or more than college), self-defined race (Black, Chinese, Japanese or White), menstrually-defined MT stage (premenopausal [regular menses], early perimenopausal [menses within the prior 3 months but less predictable], late perimenopausal [at least 3 months but less than 12 consecutive months of amenorrhea], postmenopausal [12 or more months without menses]), surgically postmenopausal [bilateral oophorectomy with our without hysterectomy prior to menopause], number of months since final menstrual period (FMP [postmenopausal women only]), current hormone therapy use (yes/no), or use of osteoporosis medication (yes/no). FMP date was the month and year at which 12 months of amenorrhea commenced or the date of surgery in the case of surgical menopause. We measured weight (kilograms) and height (meters) using calibrated scales and stadiometers; body mass index (BMI, [weight in kilograms/(height in meters) ^2^]) was calculated. Because vertebral morphometry baseline years varied, we drew information about baseline characteristics from the SWAN visit at which the initial VM was done. We used each participant’s initial spine and femoral neck BMD (i.e., first one done in SWAN) as the BMD exposures, to avoid false elevation of spine BMD values by vertebral deformity. To define never-users of major bone active medications (hormone therapy or osteoporosis medications) we considered all SWAN visits, from parent study baseline through the second VM ascertainment visit.

### Data Analysis

We computed the number of evaluable vertebral bodies at each spinal level. We employed simple linear regression to test whether the number of spinal levels visualized varied by BMI. Baseline frequencies of vertebral deformities were tabulated by spinal level and by deformity grade; un-evaluable levels were coded as no deformity present. We calculated the prevalence of any vertebral deformity (SQ grade ≥ 1) as a proportion ([number of participants with any deformity/ baseline sample size]*100); 95% Confidence Intervals (CI) were computed. We estimated prevalence for the entire sample and stratified by selected participant characteristics. For bivariate analyses, tertile cut-points for BMI and BMD were defined separately for each racial group because distributions of these characteristics differ greatly by ethnic/racial group.[20] Prevalence estimates by the presence or absence of each characteristic were compared using Chi-square test or Fisher’s exact test. We calculated incident deformity (any increase in SQ grade) per person year (PY) of observation, standardized to 1000 person-years; we also computed incidence rates stratified by age at baseline <55 years or ≥55 years. Among participants who experienced a deformity, the exact date of deformity occurrence was unknowable; we therefore censored their person-time at the midpoint of their observation period. We constructed multivariable regression models to examine the relations between prevalent deformity and BMD, race, age, BMI, and MT stage; covariates were included a priori based on prior literature.[1,2,21] We ran separate models for spine and femoral neck BMD. All analyses were done using SAS version 9.4 (SAS Institute, Cary NC). P values less than or equal to 0.05 were considered statistically significant; we did not adjust for multiple comparisons.

## Results

The VM baseline analytic sample consisted of 1446 women: 384 Black, 175 Chinese, 203 Japanese and 684 White. At baseline, mean age was 54 years (standard deviation, [SD] 2.7 years) and average BMI was 28 kg/m2 (SD, 6.8 kg/m2). The percentages of women who were premenopausal, early perimenopausal, late perimenopausal, naturally menopausal, or surgically postmenopausal were 1%, 15%, 9%, 64%, and 8% respectively; 3% of the sample had an undeterminable menopause stage. At vertebral morphometry baseline, 10% of women were using hormone therapy and 6% were taking a prescription medication for osteoporosis. Since the beginning of SWAN, 39% had ever used hormones and 8% had ever used osteoporosis medications; 5% of women had ever used both hormones and osteoporosis medications.

As expected, the most cranial portion of the VM image produced the lowest proportions of readable vertebrae: considering levels T4 through T6, between 43% and 63% of vertebral bodies could be evaluated [**Table 1**]. The commonest reasons for unreadable levels were: level not in scan, poor signal-to-noise ratio, and overlying ribs (data not shown). A greater amount of soft tissue thickness deteriorates signal-to-noise ratio; congruently, simple linear regression demonstrated that greater BMI was associated with lesser number of readable vertebral levels (B = -0.088, p<0.0001). There were 51 prevalent vertebral deformities in 46 participants; 3 participants had 2 deformities and 1 had 3 deformities [**Table 1**]. Two-thirds of the deformities were Grade 1. The vertebral levels most affected by prevalent deformities were T11 through L2.

**Table 1:** Prevalent Vertebral Deformities by Vertebral Level, Among Participants in the Study of Women’s Health Across the Nation (SWAN) Vertebral Morphometry Study (N = 1446).

Vertebral Level	Number of Vertebrae Evaluated	Number Evaluated as Percent of Maximum^1^	Number of Grade 1 Deformities	Number of Grade 2 Deformities	Number of Grade 3 Deformities	Any Deformity^2^	Percent of Total Deformities at Each Level^3^
T4	620	43%	0	0	0	0	0%
T5	778	54%	2	1	0	3	6%
T6	914	63%	2	0	1	3	6%
T7	1045	72%	2	1	0	3	6%
T8	1211	84%	3	0	0	3	6%
T9	1308	90%	2	0	1	3	6%
T10	1368	95%	1	0	0	1	2%
T11	1394	96%	7	0	1	8	16%
T12	1417	98%	6	4	0	10	20%
L1	1428	99%	6	3	1	10	20%
L2	1424	98%	1	3	1	5	10%
L3	1419	98%	2	0	0	2	4%
L4	1386	96%	0	0	0	0	0%
Deformities in All Levels (% of Total)	N/A	N/A	34 (67%)	12 (23%)	5(10%)	51(100%)	N/A

^1^ Number evaluated as percent of maximum possible: (number of evaluable vertebrae at each level/1446)*100

^2^ 46 individuals had at least one prevalent vertebral deformity. Of these, 3 had 2 deformities and 1 had 3 deformities, resulting in a total of 51 deformities among 46 women.

^3^ Percent of the total number deformities observed at each vertebral level: (number of deformities at level/51)*100

In the entire vertebral morphometry baseline sample of 1446 women, there were 46 with a vertebral deformity, resulting in a prevalence of 3.2% (95% CI, 2.3, 4.1). In the age range represented (49–62), prevalence estimates did not vary significantly by age greater than or less than 55 years (prevalence estimates 3.3% and 3.0%, respectively, p = 0.81).Bivariate analyses of characteristics potentially associated with prevalent deformity were restricted to the sub-sample of 824 women who had not taken bone active medications since SWAN baseline (hormone therapy and/or osteoporosis treatments). There were 27 women with a prevalent deformity in this sub-group **[Table 2].** There was a trend towards greater prevalence with older age (p = 0.06) and longer time since FMP (0.09). Vertebral deformity also appeared to be least prevalent in Black women, but there was not a statistically significant difference in prevalence among racial groups (p = 0.63). In bivariate analyses, other characteristics examined were not statistically significantly related to prevalence of deformity.

**Table 2:** Point Prevalence of Any Vertebral Deformity Among Participants in the Study of Women’s Health Across the Nation (SWAN) Vertebral Morphometry Sample by Participant Characteristics, Restricted to Never-Users of Bone Active Medications^1^ (N = 824).

Characteristic	Number of Participants	Number with any Deformity	Prevalence per 100 Persons (95% CI)	P-value^2^
All Women in Sample	824	27	3.3 (2.1–4.5)	
Age^3^				
< 55 (0)	482	11	2.3% (0.9–3.6)	0.06
≥55 (1)	342	16	4.7% (2.4–6.9)	
Race				
Black	241	5	2.1% (0.26–3.9)	0.63
Caucasian	349	13	3.7% (1.7–5.7)	
Chinese	117	4	3.4% (0.1–6.7)	
Japanese	117	5	4.3% (0.6–8.0)	
Menopause Transition Stage				
Premenopause	15	1	6.7% (0.0–21.0)	0.71
Early Perimenopause	172	7	4.1% (1.1–7.1)	
Late Perimenopause	99	3	3.0% (0.0–6.5)	
Natural menopause	502	16	3.2% (1.7–4.7)	
Surgical menopause	35	0	0.0%(0.0–0.0)	
Years Since FMP^4^				
< 2.9	263	5	1.9% (0.2–3.6)	0.09
≥ 2.9	239	11	4.6% (1.9–7.3)	
Tertiles of BMI ^5^				
1	279	7	2.5% (0.7–4.4)	0.64
2	264	9	3.8% (1.2–5.6)	
3	281	11	3.7 (1.6–6.2)	
Tertiles of Lumbar				
Spine BMD (g/cm2) ^5^				
1	254	11	4.3% (1.8–6.9)	0.41
2	268	9	3.4% (1.2–5.5)	
3	302	7	2.3% (0.6–4.0)	
Tertiles of Femoral Neck				
BMD (g/cm2)^5^				
1	245	12	4.9% (2.2–7.6)	0.07
2	283	4	1.4% (0.3–3.0)	
3	295	11	3.7% (1.6–5.9)	

^1^Women who did not use bone active medications (hormone therapy and/or bisphosphonates) since SWAN baseline.

^2^Chi-square p-value (Fisher’s exact test used in cases of small cell sizes).

^3^Stratified by mean age (55 years) at vertebral morphometry baseline.

^4^Stratified by mean number of years since final menstrual period (FMP) among those who had had an FMP at the time of the baseline vertebral morphometry measurement.

^5^Tertiles are defined from lowest (1) to highest (3). For univariate analyses, tertile cut-points for body mass index (BMI) and bone mineral density (BMD) were defined separately for each racial group.

The relations between prevalent deformity and BMD, race, age, BMI, and MT stage were examined in multivariable analyses; separate models used LS BMD [**Table 3**] or FN BMD [**Table 4**] as predictors. LS BMD was strongly, independently, related to prevalent deformity: the relative odds of deformity increased by 61% per SD decrement in SWAN baseline BMD (p = 0.02). We also observed an age effect, with the relative odds of deformity increasing by 21% per year increment in age (p = 0.02). The model using FN BMD as the bone density exposure revealed results similar to those of the LS model. There was a higher prevalence of deformity in relation to lower FN BMD; for each standard deviation decrement in FN BMD, the relative odds of deformity were 67% higher (p = 0.04). And, the relative odds of deformity climbed by 20% per greater year of age (p = 0.02). In both models, greater BMI was marginally statistically significantly associated with higher odds of deformity, with a relative increase of 5% per standard deviation increment in BMI (p = 0.06 in FN model).

**Table 3:** Cross Sectional Association Between Baseline Lumbar Spine Bone Mineral Density (LS BMD) and Prevalent Vertebral Deformity Adjusted for Age, Body Mass Index, Race, Baseline LS BMD and Menopause Transition Stage, the Study of Women’s Health Across the Nation (N = 823).

Characteristic	Odds Ratios of Vertebral Deformity (95% Confidence Interval)	p-value
LS BMD^1^	1.61 (1.07, 2.44)	0.024
Race^2^		0.840
Black	0.67 (0.25, 1.81)	
Chinese	1.03 (0.34, 3.16)	
Japanese	1.15 (0.41, 3.24)	
Menopause Transition Stage^3^		0.247
Late Perimenopause	0.60 (0.17, 2.12)	
Natural Postmenopause	0.36 (0.13, 1.01)	
Surgical Postmenopause	0.21 (0.12, 3.69)	
Body Mass Index^4^ (kg/m^2^)	1.04 (0.99, 1.10)	0.116
Age (years)	1.21 (1.03, 1.41)	0.022

^1^Odds of vertebral deformity are expressed per one standard deviation decrement in LS BMD; BMD was measured at SWAN study baseline. Among women who were never users of bone-active medications, mean LS BMD was 1.08g/cm^2^ (standard deviation,0.13)

^2^White race is referent.

^3^Referent group was premenopausal or early menopausal menopause transition stage; see Methods for definitions of menopause stages.

^4^Odds of vertebral deformity are expressed per one standard deviation unit of body mass index (BMI). Among women not using bone-active medications, mean BMI was 28.5 kg/m^2^ (standard deviation, 7.0).

**Table 4:** Cross Sectional Association Between Baseline Femoral Neck Bone Mineral Density (FN BMD) and Prevalent Vertebral Deformity Adjusted for Age, Body Mass Index, Race, Baseline FN BMD and Menopause Transition Stage, the Study of Women’s Health Across the Nation (N = 822).

Characteristic	Odds Ratios of Vertebral Deformity (95% Confidence Interval)	p-value
FN BMD^1^	1.66 (1.02, 2.73)	0.042
Race^2^		0.956
Black	0.78 (0.28, 2.14)	
Chinese	0.97 (0.32, 2.99)	
apanese	1.09 (0.38, 3.08)	
Menopause Transition Stage^3^		0.218
Late Perimenopause	0.63 (0.18, 2.20)	
Natural Postmenopause	0.35 (0.13, 0.98)	
Surgical Postmenopause	1.19 (0.01, 3.40)	
Body Mass Index^4^ (kg/m^2^)	1.06 (0.99, 1.12)	0.060
Age (years)	1.20 (1.02, 1.40)	0.025

^1^Odds of vertebral deformity are expressed per one standard deviation decrement in FN BMD; BMD was measured at SWAN study baseline. Among women who were never users of bone-active medications, mean FN BMD was 0.85g/cm^2^ (standard deviation 0.13)

^2^White race is referent.

^3^Referent group was premenopausal or early menopausal menopause transition stage; see Methods for definitions of menopause transition stages.

^4^Odds of vertebral deformity are expressed per one standard deviation unit of body mass index (BMI). Among women not using bone-active medications, mean BMI was 28.5 kg/m^2^ (standard deviation, 7.0)

Of the 1446 women in the baseline sample, 1108 (77%) had a follow-up vertebral morphometry scan. The earliest date of a baseline vertebral morphometry observation was 5/58/2004 and the latest date of a follow-observation was 2/8/2013; thus, the longitudinal component of the vertebral morphometry study spanned 104 calendar months and yielded 7,583 person-years (PY) of follow-up. On average, women were observed for 6.8 years (SD 0.5 years, range 5.1–8.3 years). Incident deformities of any grade were identified in 15 women, for an incidence rate of 1.98 vertebral deformities per 1000 PY (95% CI: 0.98–2.98 per 1000 PY). Among women younger than 60 years of age at VM baseline, 4 incident deformities occurred during 2321 person-years (incidence 1.72 per 1000 PY; 95% CI: 0.03–3.41 per 1000 PY). Women aged 60 years or greater at baseline experienced 11 new deformities during 5262 person-years of observation (incidence 2.09 per 1000 PY; 95% CI: 0.86–3.32 per 1000 PY). Of the 1108 women in the follow-up sample, 628 had never used bone active medications. Twelve of the fifteen incident deformities occurred in this group, yielding an incidence of 2.81 per 1000 PY during 4,263 PY of observation (95% CI: 1.22–4.41 per 1000 PY).

## Discussion

The SWAN vertebral morphometry study ascertained prevalent and incident vertebral deformities using DXA in a large, multiethnic, well-characterized, community dwelling sample of US women with an average age of 54 (+ 2.7) years at the vertebral morphometry study baseline. Vertebral deformity prevalence and incidence were low, at 3.2% and 2 per 1000PY, respectively. In SWAN, DXA’s ability to successfully image cranial-most vertebral levels proved limited: between 40% and 60% of vertebral bodies were unreadable as vertebral levels ascended from T6 to T4. With greater BMI, the number of readable vertebrae diminished.

In multivariable analyses, for each year increment in age, the relative odds of fracture increased by 20% and each standard deviation decrement in LS or FN BMD was associated with a relative increment in the odds of vertebral deformity of 60% and 67%, respectively. Each standard deviation increment in BMI was marginally statistically associated with a relative increase in the odds of deformity of 5%. The rarity of incident deformities in this sample precluded analysis of their risk factors.

Understanding the performance characteristics of VM imaging using DXA is central to the interpretation of our findings and to consideration of the use of this technology in large cohorts in general. Because we used DXA, vertebral deformity in this study is likely underestimated, particularly grade 1 (which constituted about 2/3 of the deformities in SWAN) and those occurring in the more cranial thoracic levels. A validation study in 161 postmenopausal women that compared deformity readings using Hologic 4500A DXA technology to radiographs (the gold standard) reported that DXA was 68% sensitive to the presence of any deformity and that sensitivity rose to 77% when only deformities of grade 2 or more were considered.[22] False negative vertebral morphometry readings using DXA are partly attributable to difficulty visualizing levels above T7: in the same validation, adequate visualization through T4 was only achievable in 71% of cases, whereas in 96% of instances levels up to T7 were well-imaged. Of vertebral deformities diagnosed by radiographs, 11% were in levels not ascertained by DXA.[22] To our knowledge, SWAN is the first large, longitudinal US cohort to use DXA for vertebral morphometry readings and only one of three cohort studies to have done so.[8,12] Of the other two, the Japanese Population-based Osteoporosis (JPOS) Cohort Study did not report the frequency of unreadable levels while the Tromsø Study successfully imaged greater than 95% of vertebral bodies at all levels except for the most cranial, T4, which had an 81% readability rate.[8,12] Tromso study’s high imaging success rate compared to SWAN’s may reflect Tromsø’s average BMI, which was one unit lower than that of SWAN. Thus, there is a trade-off between DXA’s attributes for epidemiological studies (low cost, high accessibility, low radiation and, consequently, the capacity for repeated examinations) vs. its lower imaging success rate and sensitivity when compared with X-rays. Whether other large studies’ experiences with DXA VM assessment will be more like SWAN’s or Tromsø’s awaits elucidation.

SWAN’s vertebral deformity prevalence of 3.2% (95%CI: 2.3, 4.1) among women in their 50’s compares favorably with estimates from 8 other studies that included similarly-aged women, 6 of which employed radiographs and 2 of which used DXA to assess deformities **[Table 5].** Prevalence estimates in the 50–59 year old age category ranged from 2.7% to approximately 12% but most samples sizes in this age stratum were between 100 and 200 in size.[8,11,23] Table **5** also illustrates that deformity prevalence is sensitive to the reading method: in 2 studies that read radiographs using 2 distinct methods, the Eastell criteria resulted in 50 to 100 percent higher values than did the Black or the McCloskey criteria.[11,24]

**Table 5:** Prevalence of Vertebral Deformities Among Women in Selected Community and Population-Based Studies^1^^,^^2^^,^^3^.

Author Year	Study	Imaging Technique and Deformity Grading Method	Age Strata and Sample Size		Number of Fractures per Stratum	Prevalence Estimate (95% CI) per 100 persons per stratum
Melton 1993	Rochester Epidemiology Project, Minnesota	Radiographs Melton [1989]	50–54	106	11	10.4 (NR)
			55–59	137	16	11.7 (NR)
			60–64	112	14	12.5 (NR)
			65–69	107	18	16.8 (NR)
Ross 1995	Hawaii Osteoporosis Study (HOS) Adult Health Study (AHS), Hiroshima	Radiographs Ross [1993]	*Hawaii*			
			50–54	1	NR	0 (NR)
			55–59	15	NR	0 (NR)
			60–64	102	NR	1.0 (NR)
			65–70	313	NR	6.1 (NR)
			*Japan*			
			50–54	56	NR	5.4 (NR)
			55–59	147	NR	4.1 (NR)
			60–64	224	NR	4.9 (NR)
			65–69	159	NR	8.2 (NR)
Tsai 1996	Taiwan Population Sample	Radiographs Eastell [1992]	50–54	88	3	4.5 (0.2, 8.8)
			55–59	83	4	4.8 (0.2, 9.4)
			60–64	104	7	6.7 (1.9, 11.5)
			65–70	618	86	13.9 (11.6, 16.3)
O’neill 1996	European Vertebral Osteoporosis Study	Radiographs Eastell [1992] McCloskey [1993]	*McCloskey Method*			
			50–54	NR	NR	5.0 (NR)
			55–59	NR	NR	7.6 (NR)
			60–64	NR	NR	9.9 (NR)
			65–69	NR	NR	13.4 (NR)
			*Eastell Method*			
			50–54	NR	NR	11.5 (NR)
			55–59	NR	NR	14.6 (NR)
			60–64	NR	NR	16.8
			65–69	NR	NR	23.5
Ling 2000	Beijing Osteoporosis Project	Radiographs^4^Black [1991] Eastell [1991]	*Black Method*			
			50–59	100	NR	3.9 (0.2, 7.7)
			60–69	100	NR	10.5 (4.6, 16.3)
			*Eastell Method*			
			50–59	100	NR	4.9 (0.7, 91)
			60–69	100	NR	16.2 (9.2, 23.2)
Kadowki2010	Japanese Population-Based Osteoporosis Cohort Study (JPOS)	DEXA^5^ McCloskey-Kanis [1993]	50–59	260	7	2.7 (NR)
			60–69	246	34	13.8 (NR)
			70–79	1206	36	17.5 (NR)
Waterloo2012	The Trøsmo Study	DEXA^6^ Kim semi-quantitative [Kim 2004]	38–60	412	14	3.4 (NR)
			60–69	721	80	11.1 (NR)
			70–87	548	105	192. (NR)
Sanfelix-Gimeno2013	Population-Based Study in Valencia, Spain (FRAVO)	Radiographs Genant semi-quantitative [Genant 1993]	50–54	118	5	4.2 (NR)
			55–59	153	11	8.7 (NR)
			60–64	169	20	15.9 (NR)
			65–70	166	24	19.1 (NR)

1 Studies tabulated are those that reported prevalence by 5- or 10- year age stratum in middle-aged women

^2^Except for semi-quantitative criteria, all vertebral deformity methods used a criterion of ≥3 standard deviation decrements from a referent standard (referent standards vary among

studies)

^3^NR = data not reported

^4^Investigators report that they sampled “approximately 100” in each stratum

^5^Hologic 4500A QDR with bone morphometric software

^6^Lunar prodigy

In the models that examined the relations between LS or FN BMD to deformities, we found that lesser LS and FN BMD were independently, statistically significantly associated with greater risk of prevalent deformity. The similarity of risk gradients evident at the spine and hip sites may appear to contradict prior work that finds a substantively lower gradient of fracture risk conferred by spine compared to hip bone density.[25] However, LS BMD in our middle-aged sample is likely to be less confounded by the age-related, pervasive degenerative disease artifacts that degrade our ability to read a true BMD signal.[26–28] We previously reported that LS but not FN BMD predicted fracture during the menopausal transition.[29]

That higher BMI is related to slightly greater risk of prevalent deformity may at first seem counterintuitive, as greater BMI is generally regarded as fracture-preventive due to its association with higher BMD.[30–32] However, when viewed from an integrated bone strength perspective, there is a pleiotropic effect of obesity: greater BMI increases BMD but not enough to compensate for the increased load on the bone.[33] Thus, adjustment for BMD (the protection pathway) exposes the deleterious pathway from BMI to vertebral deformity (which is likely to be related to the increased load); additionally, this effect is likely underestimated, because higher BMI resulted in fewer number of readable vertebral levels and likely a systematic underestimation of deformities.

The principal strengths of this study consist of its sample size of 1446 mid-life women, including women of 4 races; to our knowledge this the first vertebral deformity study with these attributes. Our large sample allowed SWAN to: 1) estimate prevalent vertebral deformity in women aged 50–60 years (prior studies, reviewed in Table **5**, had smaller samples in this age range and either did not calculate CI’s or had broader ones); and 2) to examine risk factors for fracture in this young age range. This study augments the research community’s relatively limited experience using DXA-based vertebral morphometry in a large cohort.[8,12] We used BMD values from the initial SWAN scans; while this does not guarantee that spine BMD was not falsely elevated by prevalent deformity or degenerative disease, it substantially reduces its likelihood. One limitation of the SWAN vertebral morphometry study is sub-optimal ascertainment of cranial vertebral levels; however, this is expected constraint of the technique, as reported in the VM validation[19]. Nonetheless, we do not believe that there is a substantial negative bias in our estimates of deformity because prevalence surveys, in which vertebral fractures were measured by standard x-rays, report that fractures at the levels of T4, T5 and T6 are very rare. [9,10,12] A second limitation is the small number of prevalent and incident deformities, which constrain our ability to perform relational analysis of factors related to them; this limitation is inherent in the age range of our sample. Our multivariable analyses could only be done cross-sectionally, as there were too few incident deformities to support models.

## Conclusion

Using DXA-based vertebral morphometry, we found that the prevalence of vertebral deformity in women aged 50–60 years enrolled in SWAN was low, at 3.5%, and the majority of deformities were grade 1. In cross-sectional analyses, lower bone density at both the LS and FN was most strongly related to prevalent vertebral deformity in middle-aged women, but older age and higher BMI were also associated with deformities. Approximately half of vertebral levels from T4-T6 were not imaged by DXA, but due to the rarity of fractures at these levels, this should not have a meaningful effect on our estimates. Given this technology’s known insensitivity to grade 1 deformity, we may have underestimated the prevalence and incidence of grade 1 deformities in our sample. Prospective analyses showed a low incidence of vertebral deformity, estimated at 1.7 per 1000 PY

## References

[1] Cummings SR, Melton LJ. Epidemiology and outcomes of osteoporotic fractures. Lancet. 2002 5 18;359(9319):1761–7. 12049882 10.1016/S0140-6736(02)08657-9

[2] Ensrud KE. Epidemiology of fracture risk with advancing age. J Gerontol A Biol Sci Med Sci. 2013 10;68(10):1236–42. 10.1093/gerona/glt092 23833201

[3] Cooper C, Melton LJ. Vertebral fractures. BMJ. 1992 3 28;304(6830):793–4. 1392705 10.1136/bmj.304.6830.793 PMC1881670

[4] Fink HA, Milavetz DL, Palermo L, Nevitt MC, Cauley JA, Genant HK, et al; Fracture Intervention Trial Research Group. What proportion of incident radiographic vertebral deformities is clinically diagnosed and vice versa? J Bone Miner Res. 2005 7;20(7):1216–22. Epub 2005 Mar 21. 15940375 10.1359/JBMR.050314

[5] Genant HK, Wu CY, van Kuijk C, Nevitt MC. Vertebral fracture assessment using a semiquantitative technique. J Bone Miner Res. 1993 9;8(9):1137–48. 8237484 10.1002/jbmr.5650080915

[6] Lewiecki EM, Laster AJ. Clinical review: Clinical applications of vertebral fracture assessment by dual-energy x-ray absorptiometry. J Clin Endocrinol Metab. 2006 11;91(11):4215–22. 16940447 10.1210/jc.2006-1178

[7] Cooper C, Melton LJ 3rd. Vertebral fractures. BMJ. 1992 6 20; 304(6842): 1634–5. 10.1136/bmj.304.6842.1634-b PMC1881989 1385749

[8] Kadowaki E, Tamaki J, Iki M, Sato Y, Chiba Y, Kajita E, et al Prevalent vertebral deformity independently increases incident vertebral fracture risk in middle-aged and elderly Japanese women: the Japanese Population-based Osteoporosis (JPOS) Cohort Study. Osteoporos Int. 2010 9;21(9):1513–22. Epub 2009 Nov 19. 10.1007/s00198-009-1113-9 19924494

[9] Ross PD, Fujiwara S, Huang C, Davis JW, Epstein RS, Wasnich RD, et al Vertebral fracture prevalence in women in Hiroshima compared to Caucasians or Japanese in the US. Int J Epidemiol. 1995 12;24(6):1171–7. 8824859 10.1093/ije/24.6.1171

[10] Tsai K, Twu S, Chieng P, Yang R, Lee T. Prevalence of vertebral fractures in Chinese men and women in urban Taiwanese communities. Calcif Tissue Int. 1996 10;59(4):249–53. 8781047 10.1007/s002239900118

[11] O'Neill TW, Felsenberg D, Varlow J, Cooper C, Kanis JA, Silman AJ. The prevalence of vertebral deformity in european men and women: the European Vertebral Osteoporosis Study. J Bone Miner Res. 1996 7;11(7):1010–8. 8797123 10.1002/jbmr.5650110719

[12] Waterloo S, Ahmed LA, Center JR, Eisman JA, Morseth B, Nguyen ND, et al Prevalence of vertebral fractures in women and men in the population-based Tromsø Study. BMC Musculoskelet Disord. 2012 1 17;13:3 PMCID: PMC3273434 10.1186/1471-2474-13-3 22251875 PMC3273434

[13] Sanfélix-Gimeno G, Sanfelix-Genovés J, Hurtado I, Reig-Molla B, Peiró S. Vertebral fracture risk factors in postmenopausal women over 50 in Valencia, Spain. A population-based cross-sectional study. Bone. 2013 1;52(1):393–9. Epub 2012 Oct 26. 10.1016/j.bone.2012.10.022 23103928

[14] Cauley JA, Palermo L, Vogt M, Ensrud KE, Ewing S, Hochberg M, et al Prevalent vertebral fractures in black women and white women. J Bone Miner Res. 2008 9;23(9):1458–67. PMCID: PMC2683160 10.1359/jbmr.080411 18442309 PMC2683160

[15] Adachi JD, Loannidis G, Berger C, Joseph L, Papaioannou A, Pickard L, et al; Canadian Multicentre Osteoporosis Study (CaMos) Research Group. The influence of osteoporotic fractures on health-related quality of life in community-dwelling men and women across Canada. Osteoporos Int. 2001;12(11):903–8. 11804016 10.1007/s001980170017

[16] Ling X, Cummings SR, Mingwei Q, Xihe Z, Xioashu C, Nevitt M, et al Vertebral fractures in Beijing, China: the Beijing Osteoporosis Project. J Bone Miner Res. 2000 10;15(10):2019–25. 11028456 10.1359/jbmr.2000.15.10.2019

[17] Klotzbuecher CM, Ross PD, Landsman PB, Abbott TA III, Berger M. Patients with prior fractures have an increased risk of future fractures: A summary of the literature and statistical synthesis. J Bone Miner Res. 2000 4; 15(4):721–739. 10780864 10.1359/jbmr.2000.15.4.721

[18] Sowers MF.; Crawford SL.; Sternfeld B., et al SWAN: A multiethnic, community-based cohort study of women and the menopausal transition In: Lobo RA.; Kelsey J.; Marcus R., editors. Menopause: Biology and pathobiology. San Diego: Academic Press; 2000

[19] Schousboe JT, Vokes T, Broy SB, Ferrar L, McKiernan F, Roux C, et al Vertebral Fracture Assessment: the 2007 ISCD Official Positions. J Cli Densitom. 2008 Jan-Mar;11(1): 92–108. 10.1016/j.jocd.2007.12.008 18442755

[20] Finkelstein JS, Brockwell SE, Mehta V, Greendale GA, Sowers MR, Ettinger B, et al Bone mineral density changes during the menopause transition in a multiethnic cohort of women. J Clin Endocrinol Metab. 2008 3;93(3):861–8. Epub 2007 Dec 26. 18160467 10.1210/jc.2007-1876 PMC2266953

[21] Hosmer DW, Lemeshow S. Multiple logistic regression John Wiley & Son, Inc., 2000.

[22] Rea JA, Li J, Blake GM, Steiger P, Genant HK, Fogelman I. Visual assessment of vertebral deformity by X-ray absorptiometry: a highly predictive method to exclude vertebral deformity. Osteoporos Int. 2000;11(8):660–8. 11095168 10.1007/s001980070063

[23] Melton LJ 3rd, Kan SH, Frye MA, Wahner HW, O'Fallon WM, Riggs BL. Epidemiology of vertebral fractures in women. Am J Epidemiol. 1989 5;129(5):1000–11. 2784934 10.1093/oxfordjournals.aje.a115204

[24] Ling X, Cummings SR, Mingwei Q, Xihe Z, Xioashu C, Nevitt M, et al Vertebral fractures in Beijing, China: the Beijing Osteoporosis Project. J Bone Miner Res. 2000 10;15(10):2019–25. 11028456 10.1359/jbmr.2000.15.10.2019

[25] Leslie WD, Lix LM, Tsang JF, Caetano PA; Manitoba Bone Density Program. Single-Site vs Multisite Bone Density Measurement for Fracture Prediction. Arch Intern Med. 2007;167(15):1641–7. 17698687 10.1001/archinte.167.15.1641

[26] Jones G, Nguyen T, Sambrook PN, Kelly PJ, Eisman JA. A longitudinal study of the effect of spinal degenerative disease on bone density in the elderly. J Rheumatol. 1995 5;22(5):932–6. 8587085

[27] von der Recke P, Hansen MA, Overgaard K, Christiansen C. The impact of degenerative conditions in the spine on bone mineral density and fracture risk prediction. Osteoporos Int. 1996;6(1):43–9. 8845599 10.1007/BF01626537

[28] Liu G, Peacock M, Eilam O, Dorulla G, Braunstein E, Johnston CC. Effect of osteoarthritis in the lumbar spine and hip on bone mineral density and diagnosis of osteoporosis in elderly men and women. Osteoporos Int 1997;7 (6) 564– 569. 9604053 10.1007/BF02652563

[29] Cauley JA, Danielson ME, Greendale GA, Finkelstein JS, Chang YF, Lo JC, et al Bone resorption and fracture across the menopausal transition: the Study of Women's Health Across the Nation. Menopause. 2012 11;19(11):1200–7. 10.1097/gme.0b013e31825ae17e 22850443 PMC3483443

[30] De Laet C, Kanis JA, Oden A, Johanson H, Johnell O, Delmas P, et al Body mass index as a predictor of fracture risk: a meta-analysis. Osteoporos Int. 2005; 16:1330–8. 15928804 10.1007/s00198-005-1863-y

[31] Looker AC, Flegal KM, Melton LJ 3rd. Impact of increased overweight on the projected prevalence of osteoporosis in older women. Osteoporos Int. 2007; 18:307–13. 17053871 10.1007/s00198-006-0241-8

[32] Felson DT, Zhang Y, Hannan MT, Anderson JJ. Effects of weight and body mass index on bone mineral density in men and women: the Framingham study. J Bone Miner Res. 1993; 8:567–73. 8511983 10.1002/jbmr.5650080507

[33] Ishii S, Cauley JA, Greendale GA, Nielsen C, Karvonen-Gutierrez C, Ruppert K, et al Pleiotropic effects of obesity on fracture risk: the Study of Women's Health Across the Nation. J Bone Miner Res. 2014 12;29(12):2561–70 10.1002/jbmr.2303 24986773 PMC4403760

